# Analysis of risk factors for hospital-acquired pneumonia in schizophrenia

**DOI:** 10.3389/fpsyt.2024.1414332

**Published:** 2024-08-16

**Authors:** Yu-hang Chen, Cong-ying Ren, Yu Liao

**Affiliations:** ^1^ Department of Operations Management, Chongqing Mental Health Center, Chongqing, China; ^2^ Department of Hospital Infection Control, Chongqing Mental Health Center, Chongqing, China; ^3^ Cardiology Department, People’s Hospital of Chongqing Rongchang District, Chongqing, China

**Keywords:** schizophrenia, pneumonia, hospital-acquired pneumonia, risk factors, hospital-acquired infection

## Abstract

**Background:**

Hospital-acquired pneumonia is one of the most important causes of recurrent illness, disease progression, and even death during hospitalization. Patients with schizophrenia have the special characteristics of their disease, and at the same time, the occurrence of hospital-acquired pneumonia is more common among patients with schizophrenia due to the prolonged stay in closed wards, accompanied by various factors such as age, gender, and nutritional status.

**Methods:**

The PubMed, Web of Science, Cochrane Library, China National Knowledge Infrastructure (CNKI), and China Biomedical Literature Database (CBM) databases were searched with a timeframe of build to February 2024 to collect studies on factors influencing hospital-acquired pneumonia in patients with schizophrenia. Two researchers independently screened the literature, extracted data, and analyzed them.

**Results:**

A total of 5 papers including 85246 patients were included in the literature, which suggested that benzodiazepines (especially the use of clozapine), combination of antipsychotics, mood stabilizers, modified electroconvulsive therapy (MECT), duration of hospitalization, underlying diseases, hyperglycemia, and salivation/dysphagia were important risk factors for hospital-acquired pneumonia in schizophrenia patients, and that advanced age, smoking and alcohol drinking Older age, smoking and drinking habits, malnutrition, and underlying diseases are also risk factors for hospital-acquired pneumonia.

**Conclusions:**

Patients with schizophrenia are at a higher risk of developing hospital-acquired pneumonia, so identifying the risk factors associated with hospital-acquired pneumonia and evaluating them comprehensively and promptly during hospitalization facilitates the development of early interventions, which are essential for improving the prognosis of patients with schizophrenia.

## Introduction

Schizophrenia (SCZ), as a serious mental disorder, usually manifests clinically with a range of psychiatric symptoms, such as hallucinations, delusions, emotional apathy, and cognitive dysfunction (1, 2). According to a 2018 World Health Organization report, more than 20 million people worldwide already suffer from SCZ, and China accounts for about half of them (3). Due to its disease characteristics of high morbidity, high disability rate, and low cure rate (4), SCZ imposes heavy psychological and economic burdens on patients’ families as well as society (5, 6), and has become a major social challenge (4).

Hospital-acquired pneumonia (HAP) is defined as pneumonia that is not in the incubation period at the time of admission and occurs more than 48 hours after admission (7, 8). Previous studies have shown a high incidence of HAP in Chinese patients with mental disorders, with a large psychiatric specialty hospital in Sichuan reporting a 7.8% incidence of HAP in middle-aged and elderly (≥50 years old) patients with schizophrenia (9), and Taiwan’s Specialized Medical Institutes for Mental Disorders similarly noting an incidence of HAP in patients with severe mental disorders of 14.7/1,000 persons/year (10). HAP increases the treatment costs, prolongs hospitalization, and leads to significant increases in patient morbidity and mortality (11), thus early identification of the risk of HAP in patients with schizophrenia and timely interventions to reduce the adverse effects of HAP are critical.

## Materials and methods

Five databases, PubMed, Web of Science, Cochrane Library, China National Knowledge Infrastructure (CNKI), and China Biomedical Literature Database (CBM), were searched by computer, and the time limit for searching was from the establishment of the databases to February 2024, and the search strategy was shown in Appendix Box 1 for PubMed. To search for studies on the factors affecting hospital-acquired pneumonia in patients with schizophrenia, the search terms in Chinese and English were pneumonia, schizophrenia, risk factors, etc. Taking PubMed as an example, the specific search strategies were as follows.

((((“Schizophrenia”[Mesh]) OR (((((Schizophrenias[Title/Abstract]) OR (Schizophrenic Disorders[Title/Abstract])) OR (Disorder, Schizophrenic[Title/Abstract])) OR (Disorders, Schizophrenic[Title/Abstract])) OR (Schizophrenic Disorder[Title/Abstract]))) AND ((“Risk Factors”[Mesh]) OR ((Factor, Risk[Title/Abstract]) OR (Risk Factor[Title/Abstract])))) AND ((“Pneumonia”[Mesh]) OR (((((((((Pneumonias[Title/Abstract]) OR (Pneumonitis[Title/Abstract])) OR (Pneumonitides[Title/Abstract])) OR (Lung Inflammation[Title/Abstract])) OR (Inflammation, Lung[Title/Abstract])) OR (Inflammations, Lung[Title/Abstract])) OR (Lung Inflammations[Title/Abstract])) OR (Pulmonary Inflammation[Title/Abstract])) OR (Inflammation, Pulmonary[Title/Abstract])))) AND ((“Case-Control Studies”[Mesh]) OR ((((((((((((((((Case-Control Study[Title/Abstract]) OR (Studies, Case-Control[Title/Abstract])) OR (Study, Case-Control[Title/Abstract])) OR (Case-Comparison Studies[Title/Abstract])) OR (Case Comparison Studies[Title/Abstract])) OR (Case-Comparison Study[Title/Abstract])) OR (Studies, Case-Comparison[Title/Abstract])) OR (Study, Case-Comparison[Title/Abstract])) OR (Case Control Studies[Title/Abstract])) OR (Case Control Study[Title/Abstract])) OR (Studies, Case Control[Title/Abstract])) OR (Study, Case Control[Title/Abstract])) OR (Cohort Studies[Title/Abstract])) OR (Cohort Study[Title/Abstract])) OR (Studies, Cohort[Title/Abstract])) OR (Study, Cohort[Title/Abstract]))).

Literature was screened, data extracted, and checked independently by 2 researchers. In case of disagreement, it was discussed and resolved with the 3rd researcher. Literature was screened using EndNote literature management software, and after initial screening by reading the titles and abstracts of the literature, the full text was further read for re-screening to determine final inclusion. Data extraction included: first author, year of publication, sample size, risk factors, OR, and 95% CI.

## Results

A total of 102 publications were obtained from the initial review, including PubMed (n=12), Cochrane Library (n=20), Web of Science (n=67), CNKI (n=1), and CBM (n=2). Five studies were included after a cascade screening process.

According to the relevant studies included in the literature, the results showed that the use of benzodiazepines, especially clozapine, is an important risk factor for the development of hospital-acquired pneumonia in patients with schizophrenia, and the combination of 2 or more antipsychotics induces the development of pneumonia. In addition, mood stabilizers, MECT, length of hospitalization, underlying disease, hyperglycemia, and salivation/dysphagia also contribute somewhat to the risk of hospital-acquired pneumonia. See **Table 1** for more details.

**Table 1:** Results of the analysis of risk factors for hospital-acquired pneumonia in patients with schizophrenia.

Included studies	Sample size	Risk factors	OR	p-value	95%CI
Cheng 2018 (12)	34929	benzodiazepines	Midazolam 5.54-7.77 Diazepam 2.89-4.08 Lorazepam 1.81-2.58 Triazolam 1.10-2.93	P<0.001	/
Yang 2023 (13)	7085	benzodiazepines	3.13	P<0.001	1.95-5.03
		mood stabilizers	3.33	P<0.001	1.79–6.20
		MECT	2.58	P=0.001	1.49–4.46
Kuo 2013 (14)	33024	clozapine	3.18	P<0.001	2.62-3.86
Milano 2020 (15)	872	clozapine	2.37	P=0.005	1.30–4.32
		combination of antipsychotics	2.28	P=0.022	1.13-4.62
Han 2021 (16)	9336	length of hospitalization	1.025	P<0.05	1.004-1.047
		clozapine	3.634	P<0.05	1.842-7.171
		combination of antipsychotics	2.653	P<0.05	1.380-5.100
		underlying disease	2.268	P<0.05	1.167-4.411
		salivation/swallowing Disorder	56.726	P<0.05	7.534-427.078
		hyperglycemia	4.129	P<0.05	1.032-16.520

## Discussion

### Effect of demographic characteristics on HAP

Although the above findings do not address relevant demographic factors, a series of studies have reported that age, gender, smoking, and alcohol consumption can influence the occurrence of HAP. According to Foppa et al. (17), mortality due to influenza increases with age, with the highest rate in those over 65 years of age. In a case-control study that included 7807 patients with COVID-19, Merzon et al. (18) found that the incidence of COVID-19 was associated with age over 50 years. Related studies have shown that age over 50 years is a risk factor for death from pneumonia (17, 19). Therefore, age is an important factor of concern in the progression of respiratory infections. Yang, M. (2021) et al. In a retrospective study conducted in a large psychiatric hospital from 2016 to 2020, 2,617 schizophrenia inpatients aged 50 years and older were infected with HAP in 203 cases, with an infection rate of 7.8%. There was a significant positive correlation between patient age and HAP, which may be a result of several factors such as a general weakening of the immune system, vaccine efficacy, mucosal barrier function, and cough reflex in elderly patients (9). During the hospitalization of elderly patients with schizophrenia, medical staff need to pay extra attention to the development of respiratory infections in particular, which is a weak point in medical care.

There are gender differences in the clinical presentation of patients with schizophrenia, and the differences may be related to differences in gene expression (20), brain structure and function (21), and even socio-cultural (22). It is known that there are more women than men with schizophrenia and in a study by Yang, M. (2021) et al. the proportion of men with schizophrenia in middle-aged and elderly patients was about 35.1% compared to a high of 64.9% for women. Surprisingly, however, the incidence of HAP was significantly higher in male patients than in females, which was hypothesized to be possibly related to long-term smoking habits and poorer oral self-management in males (9).

In the general population, smoking has a high level of acceptance as a risk factor for the development of pneumonia. Similarly, smoking behavior remains highly prevalent in patients with schizophrenia, and such patients are more difficult to treat for smoking cessation than the general population (23). Although it has been hypothesized that smoking cessation exacerbates associated psychiatric symptoms in patients, there is still no clear evidence to support this claim (24), and healthcare professionals should still encourage patients to quit smoking to prevent HAP. Similarly, alcohol utilization disorder (AUD) has long been recognized as a risk factor for exacerbation of respiratory diseases (25–27). This includes increased susceptibility to viral pneumonia (28–31) and the malignant outcome that causes acute respiratory distress syndrome (ARDS) (32). In particular, a study on human bronchial epithelial cells noted that alcohol dependence triggers an increase in airway epithelial cell inflammation, which further disrupts their barrier function and provides an opportunity for viral invasion, thus supporting the hypothesis of alcohol dependence as an increased risk for viral pneumonia (33). Therefore, control of alcohol intake should be equally emphasized, especially in patients with chronic alcoholism, and should be taken a detailed history and restrained.

### Effect of nutritional status, length of hospitalization, and underlying disease on HAP

Balanced nutrition can maintain autoimmunity and thus achieve self-protection of the organism against the invasion of pathogenic bacteria, so the nutritional status is closely related to the susceptibility of pathogenic bacteria. Malnutrition increases the host’s susceptibility to infection, and pathogen invasion interferes with the host’s metabolism, which in turn continues to deteriorate the host’s nutritional status (34). Body weight, a common measure of an individual’s nutritional status, is also strongly associated with the risk of pneumonia (35, 36), and a study by Takahiro et al. (37) showed that the prevalence of pneumonia in lean schizophrenic patients was 41.6%. Therefore, nutritional assessment and early intervention in schizophrenic patients need to be taken seriously during hospitalization, and improvement of body weight is an easy-to-administer therapeutic tool.

The effect of hospitalization time on HAP is accompanied by closed hospital management. Prolonged closed hospitalization is not conducive to the cultivation of patients’ social regression and social ability; patients are in a closed ward environment for a long time, due to the restriction of daily activities, poor air circulation, the hospital in and out of the personnel is more complex, and at the same time there is a situation in which patients spit in the ward and the environmental hygiene and cleaning are not in place (38, 39). Therefore, prolonged hospitalization undoubtedly increases the risk of hospital-acquired infections.

Long-term hospitalized elderly patients with schizophrenia, often suffer from two or more chronic underlying diseases at the same time, which include stroke, malignancy, respiratory diseases, hypertension, and diabetes mellitus (40). Studies (41) have shown that the impact of multiple chronic underlying diseases on an individual’s functioning, quality of life, and risk of death is greater than the sum of the individual effects of the aforementioned diseases. In particular, elderly patients with underlying respiratory diseases are more susceptible to viral pneumonia (42). The morbidity and mortality of viral pneumonia are increased in patients with comorbid underlying respiratory diseases such as asthma and chronic obstructive pulmonary disease (43–45). For patients with schizophrenia, the interaction between mental illness and multiple chronic underlying diseases not only seriously affects the prognosis of patients, but also makes it more difficult for psychiatrists to make diagnostic and therapeutic decisions.

### Effect of medication on HAP

Many studies have included the use of drugs as an important risk factor for HAP. On the one hand, it is the main treatment for schizophrenia; on the other hand, there is a wide variety of medications, which exert their effects through different mechanisms of action and are accompanied by a series of adverse drug reactions.

On the one hand, antipsychotic drugs achieve improvement of SCZ symptoms by exerting sedative and muscle relaxation effects, but on the other hand, this class of drugs simultaneously inhibits the motor function of respiratory cilia and weakens the ability of respiratory clearance of pathogenic bacteria, which leads to an increase in the risk of pneumonia and the incidence of HAP (46). However, the effects of different types of antipsychotics on pneumonia are still controversial in the medical community. One study (47) indicated that the use of first-generation antipsychotics (FGA) increased the incidence of pneumonia in hospitalized patients and led to an increased risk of death. Other studies have similarly confirmed a degree of association between second-generation antipsychotics (SGA) and the development of HAP, and in particular, clozapine, which is a commonly used medication, has a high correlation with the risk of pneumonia (14). Especially in elderly psychiatric patients, both the application of FGA and SGA lead to an increased risk of pneumonia (46, 48), which emphasizes that age is an important factor to be taken into account when choosing a drug regimen.

According to Kuo et al, (14) drug studies suggest that the link between pneumonia and antipsychotics may be mediated by the affinity between the drugs and the muscarinic 1 receptor (M1) and histaminergic 1 receptor (H1). Antipsychotic drugs competitively bind to M1 receptors and impede the binding of M1 receptors to acetylcholine, resulting in anticholinergic effects that subsequently lead to a range of pathophysiologic responses, such as dry mouth, abnormally reduced esophageal dilatation and peristalsis, and reflux of gastric contents, which ultimately induces aspiration pneumonitis (49); at the same time, the anticholinergic effects cause an increase in mucus secretion from bronchial tubes, which results in more viscous secretions to obstruct the airways, thereby exacerbating bronchiolitis. The above studies (14, 46, 48) also further confirmed that clozapine, one of the typical representatives of SGAs, has the highest affinity for M1 receptors, and olanzapine and quetiapine have intermediate levels of affinity. Yang, M. (2021) et al. (9) reconfirmed that the SGAs (quetiapine, clozapine, and olanzapine) are associated with an increased risk of HAP by ruling out drug-drug interactions, and their risk was 1.5-1.8 times higher than without these drugs, with clozapine having the highest ratio of pneumonic risk, followed by olanzapine and quetiapine. In addition to this, SGAs (quetiapine, clozapine, and olanzapine) also have a high affinity for H1 receptors [40], which leads to excessive sedation and salivation by antagonizing H1 receptors in the central nervous system, a mechanism that further aggravates aspiration pneumonia (50). Therefore, for SCZ patients with high-risk factors, psychiatrists should minimize the dosage of antipsychotics when the patient’s condition has been effectively controlled, thus preventing the development of HAP.

Unlike SGA, FGA mediates the production of HAP by affecting dopamine receptors. It is known that blockade of dopamine receptors may lead to Extrapyramidal reactions (EPS), such as symptoms of dyskinesia, rigidity, and spasms of the oral and pharyngeal muscles, which may further lead to dysphagia and eventual development of aspiration pneumonia (51), and Takahiro et al. demonstrated that chlorpromazine, one of the FGAs, was associated with an increased risk of aspiration pneumonia at equivalent doses (37). Whereas haloperidol, which is also a representative drug of FGA, as a potent, small-dose applied antipsychotic, it is capable of rapidly controlling symptoms during acute episodes of psychiatric disorders, and its antagonistic effect on dopamine receptors is 20-40 times higher than that of chlorpromazine at the same dosage, yet the incidence of HAP was lower in the haloperidol group through the observation by Yang, M. (2021) (9). However, the results of this study were not further explained. Therefore, we need to control the choice of drug type and dose to achieve the reduction of HAP.

Although antipsychotic monotherapy is the American Psychiatric Association’s recommended guideline for the treatment of schizophrenia, combinations of antipsychotics and nonantipsychotics are more common in clinical practice (52–55), and it is more difficult to study whether nonantipsychotics as adjunctive therapy can independently trigger HAP.

Yang, M. (2021) et al. (9) showed that antipsychotic drugs combined with acetylglutamide affected the occurrence of HAP by analyzing the combination of the two drugs. Acetylglutamide mainly acts as an adjunctive treatment for neurological decline in elderly patients, which passes through the blood-brain barrier and breaks down into glutamate and γ-aminobutyric acid (GABA), which can inhibit postsynaptic neuronal excitation after binding to GABA receptors. The study concluded from the application of acetylglutamide injection that when the infusion rate is too fast or too large, the blood concentration of the drug rises rapidly, stimulating the norepinephrine neurons located in the ventral-lateral portion of the medulla oblongata, which inhibits the activity of cardiac sympathetic neurons, leading to vasodilation, a drop in blood pressure, and even hypovolemic shock, a mechanism of action that carries the risk of inducing aspiration pneumonia.

In addition to this, the combination of antipsychotics and benzodiazepines is often necessary to control acute-phase symptom exacerbations or severe psychotic relapses in patients with schizophrenia (56). Recent studies have shown that benzodiazepines are associated with an increased risk of pneumonia, Cheng et al. (12) reported a dose-dependent relationship between benzodiazepines and pneumonia in patients with schizophrenia, while a recent review also reported an association between benzodiazepines and an increased risk of pneumonia (57).

Mood stabilizers are used as a class of medications to reduce aggression or stabilize mood in the treatment of patients with schizophrenia (56). Taipale et al. (58) found that phenytoin sodium, carbamazepine, valproic acid, and pregabalin were associated with an increased risk of pneumonia, and Han et al. (59) concluded that patients with psychotic disorders treated with mood stabilizers had a higher likelihood of developing HAP.

### Effect of MECT on HAP

Modified electroconvulsive therapy (MECT) is currently recognized as an effective treatment for schizophrenia, especially in patients with drug resistance, aggression, catatonia, major depression, or suicidal behavior (60), which often causes cognitive dysfunction, headache, nausea, vomiting, mild anxiety The treatment often causes cognitive dysfunction, headache, nausea, vomiting, mild anxiety, and fever, and may also increase the incidence of pneumonia (61). Anesthetics and muscle relaxants are often used to mitigate side effects (62, 63), but there is an increased risk of aspiration. Serum cytokine concentrations are strongly associated with infections, with one review noting elevated concentrations of the tumor necrosis factors TNF-α, IL-1β, and IL-6 after a single MECT treatment (64), and another meta-analysis showing that MECT induces increased IL-6 levels and potentially decreased TNF-α levels (65).

Yang, M. (2023) et al. (66) confirmed MECT as one of the risk factors for HAP in patients through a study, noting that the incidence of HAP among MECT patients was 6.52%, suggesting that MECT treatment may have induced an acute immune-inflammatory response including elevated plasma cortisol and IL-1β or IL-6 levels while improving schizophrenia behaviors and that long-term treatment reduced blood TNF-α and IL-6 levels, leading to decreased immunity and increased patient susceptibility. In addition to this, the study noted that all patients had a very high risk of developing HAP within 1 day of receiving MECT, and thus patients should be monitored on the first day after each MECT treatment, with particular attention to clinical care after the first 3 courses of MECT. It is worth noting that the study found that male patients were more likely to develop HAP than females (approximately 2.3 times more likely in males than in females), and that the risk factors for HAP differed between the two groups.

This study has the following limitations. Firstly, the small amount of literature included and the inconsistent sample size of individual studies reduced the reliability of the findings of this study. Secondly, the type of studies included was cross-sectional, which may have caused some bias. Thirdly, the small sample size of the individual included studies may have caused some bias in the results. And finally, it is unfortunate that the influential factor of psychotherapy and counseling intervention was not included in the discussion of this study; this type of therapeutic measures as a complementary means of drug maintenance therapy can help to improve the clinical outcome of patients with schizophrenia and improve the quality of life of patients, but this study did not obtain clear data to show the protective effect of hospital-acquired infections, which is a direction of concern for our future research.

## Conclusion

In conclusion, benzodiazepines (especially clozapine), the combination of antipsychotics, mood stabilizers, nonconvulsive electroconvulsive therapy (MECT), length of hospitalization, underlying diseases, hyperglycemia, and salivation/dysphagia are important risk factors for hospital-acquired pneumonia in patients with schizophrenia, and also, advanced age, smoking and drinking habits, malnutrition, and underlying diseases are also risk factors for hospital-acquired pneumonia. The risk factors for hospital-acquired pneumonia are age, smoking and drinking habits, poor nutrition, and underlying diseases. Meanwhile, the difference in the occurrence of HAP based on gender may be due to differences in physiological conditions, immune function, and lifestyle habits. If a prediction model can be effectively established to evaluate the risk factors for HAP in SCZ patients, preventive strategies can be established promptly to guide future clinical management and nursing care, which will play a positive role in reducing the incidence of HAP in SCZ patients.
